# Ischemic stroke prediction of patients with carotid atherosclerotic stenosis *via* multi-modality fused network

**DOI:** 10.3389/fnins.2023.1118376

**Published:** 2023-02-24

**Authors:** Peng Lv, Jing Yang, Jiacheng Wang, Yi Guo, Qiying Tang, Baptiste Magnier, Jiang Lin, Jianjun Zhou

**Affiliations:** ^1^Department of Radiology, Zhongshan Hospital, Fudan University and Shanghai Institute of Medical Imaging, Shanghai, China; ^2^School of Medicine, Xiamen University, Xiamen, China; ^3^Department of Computer Science at School of Informatics, Xiamen University, Xiamen, China; ^4^Department of Radiology, Zhongshan Hospital Xiamen, Fudan University, Xiamen, China; ^5^Xiamen Municipal Clinical Research Center for Medical Imaging, Xiamen, China; ^6^Euromov Digital Health in Motion, Univ Montpellier, IMT Mines Ales, Ales, France

**Keywords:** brain MRI, carotid atherosclerotic stenosis, ischemic stroke, deep learning, diagnostic classification

## Abstract

Carotid atherosclerotic stenosis of the carotid artery is an important cause of ischemic cerebrovascular disease. The aim of this study was to predict the presence or absence of clinical symptoms in unknown patients by studying the existence or lack of symptoms of patients with carotid atherosclerotic stenosis. First, a deep neural network prediction model based on brain MRI imaging data of patients with multiple modalities is constructed; it uses the multi-modality features extracted from the neural network as inputs and the incidence of diagnosis as output to train the model. Then, a machine learning-based classification algorithm is developed to utilize the clinical features for comparison and evaluation. The experimental results showed that the deep learning model using imaging data could better predict the clinical symptom classification of patients. As part of preventive medicine, this study could help patients with carotid atherosclerosis narrowing to prepare for stroke prevention based on the prediction results.

## 1. Introduction

Ischemic stroke is a major cause of morbidity and mortality worldwide. Carotid atherosclerotic stenosis is an important risk factor of ischemic stroke and accounts for approximately 20% of stroke patients (Johnson et al., 2019). At present, it is proved that the main mechanisms of stroke due to carotid atherosclerotic stenosis are the intracranial arteries embolism caused by carotid unstable plaque rupture and the reduction of cerebral blood flow caused by moderate and severe carotid stenosis (Gupta et al., 2013). Then, cerebral ischemic injury occurs and patient presents with clinical symptoms of stroke.

Nowadays, the widely use of magnetic resonance imaging (MRI) in stroke clinical practice makes carotid atherosclerotic stenosis-related cerebral ischemic injury easily to be detected. Cerebral signal abnormality is the direct embodiment of the injury caused by carotid stenosis. Cerebral signal abnormality is much more frequent than clinical stroke and is highly prevalent in older people. Generally, the signal abnormalities of cerebral ischemic injury can present as several types on routine MRI, including (but not limited to) infarctions and white matter hyperintensities. Many studies have suggested that cerebral signal abnormalities are associated with an increased risk of ischemic stroke (Debette et al., 2019; Epstein et al., 2022). Therefore, better exploring the cerebral signal abnormalities on MRI is crucial to stroke prediction strategies in patients with carotid atherosclerotic stenosis. Best medical therapy has showed the effectiveness in reducing stroke risk (Abbott, 2009; Reiff et al., 2020). Patients with carotid stenosis who have a high possibility to suffer stroke are more likely to benefit from intensive and rigorous medical therapy.

In recent years, deep learning (DL) has been increasingly applied in the medical field to solve clinical problems. Stroke medicine is a quite suitable application for DL because vast amount and wide variety of brain imaging data need to be collected, combined and analyzed in making clinical decisions. Most of previous studies adapted DL algorithms based on brain MRI data have focused on acute ischemic stroke, either for the infarction detection and segmentation, or for the clinical prognosis evaluation (Yu et al., 2020; Li et al., 2022; Shin et al., 2022). However, few has concerned about predicting risk of ischemic stroke.

Given the clear correlation between cerebral signal abnormalities and ischemic stroke, and the advantage of DL techniques to integrated process variety of visible and invisible features, we present a DL algorithm to predict the occurrence of ischemic stroke in patients with carotid atherosclerotic stenosis using their routine brain MRI images. Deep convolutional neural networks require a large number of training parameters, which not only create a classification burden but also may lead to overfitting. We hope that the existing model can meet the clinical needs without overtraining parameters. Finally, we chose 3d Resnet-18 (He et al., 2016) because it not only uses more hidden layers and images for training, it also has a smaller convolutional kernel, which can effectively improve model performance. T1-weighted imaging (T1WI), T2-weighted imaging (T2WI), fluid attenuated inversion recovery (FLAIR), and apparent diffusion coefficient (ADC) were the most common sequences used in clinic and different modalities can provide different information. For example, T2WI and FLAIR were extremely useful to detect cerebral ischemic injury lesions because most lesions presented as hyperintensities on these two modals but often showed subtle or obvious differences. T1WI could provide good cerebral anatomical information and helped to discriminate infarctions and white matter hyperintensity. Besides, ADC was also important for diagnosing cerebral ischemic injury lesions in different stages.

Based on this consideration, we designed a channel attention mechanism to learn salient regions in different modal images. Feature fusion can enhance the ability of characterizing the prognosis of ischemic stroke in patients with carotid atherosclerotic stenosis. In addition, clinical data are used in the algorithm to improve the prediction accuracy.

## 2. Related work

To date, several stroke risk prediction models were developed, most of them were based on clinical data, especially the demographic factors and common cardiovascular risk factors (Flueckiger et al., 2018; Arafa et al., 2022; Chun et al., 2022). The Framingham Stroke Risk Score (FSRS) (Flueckiger et al., 2018) was one of the most widely used risk score for prediction of stroke. It provided sex-specific predictions of the absolute risks of total stroke in the future 5–10 years, which representing important information for the patient. The revised FSRS updated stroke risk factors prevalence and stroke rate incidence and showed better discriminative ability and calibration for incident stroke than original FSRS (Flueckiger et al., 2018).

Computational learning offers opportunity to improve accuracy by exploiting complex interactions between risk factors. Recent years, some studies have tried to employ machine learning (ML) algorithms to predict the stroke risk (Weng et al., 2017; Teoh, 2018; Penafiel et al., 2020). Weng et al. (2017) used four different algorithms including logistic regression, random forest, gradient boosting machines, and neural networks to predict the patients at risk of stroke. Teoh (2018) proposed a Recurrent Neural Network method in combination with a custom loss function to predict a diagnosis of stroke. The best neural network of the model attained an area under the curve (AUC) of 0.67. Penafiel et al. (2020) presented a model for predicting stroke occurrences within a year based on Dempster-Shafer theory. The model achieved good performance for stroke risk prediction even with some missing data.

Commonly used ML-based solutions require manual computation of grayscale features (Acharya et al., 2013a,b; Araki et al., 2017); then a training classifier is used to learn these features. Thereafter, the model is trained, features from external data can be learned to predict its class risk (Saba et al., 2019b). However, this ML-based solution is *ad-hoc*, slow, and not universal, in addition to lacking reliability and stability. In recent years, DL techniques have dominated various industries, especially in medical imaging (Biswas et al., 2019; Khanna et al., 2019; Saba et al., 2019a). This technique provides an alternative to ML strategies. It is able to automatically learn feature maps in the original image. Changes in grayscale contrast are dynamically adjusted through neural network layers of the DL architecture. Lekadir et al. (2016) developed a Convolutional Neural Network (CNN) model for the classification of plaque components by extracting 90,000 plaques from 50 *in vivo* ultrasound images and achieved a correlation coefficient of 0.90. This study aimed to develop and design an automated carotid plaque characterization and binary classification system, i.e., symptomatic and asymptomatic, with an implementation on a supercomputer through a DL framework.

Generally, single modality medical images often do not contain enough information to reach a reliable diagnosis. Clinical diagnosis often uses multiple sources of information, such as brain tumor segmentation with multiple MR images. Effective fusion of multi modal information is of great importance in the medical field for better diagnostic prediction. Mainly, CNNs use probabilistic methods for information fusion, which can be divided into three strategies:

- Image-level fusion, such as input data cross talk (Peiris et al., 2021),
- Feature-level fusion, such as attention mechanism cross talk (Zhou et al., 2020),
- Decision-level fusion, such as weighted average (Kamnitsas et al., 2017).

However, probabilistic fusion cannot effectively manage the conflicts that occur when different labels based on several modalities are assigned to the same voxel. In this paper, a feature-level fusion is used to fuse the brain MRI images of the four modalities extracted by the model with shared structures, effectively reducing the parameters while learning complementary features of different modalities.

## 3. Method

### 3.1. Dataset

In this dataset, 282 patients are included (mean age 69.8 ± 9.4; 250 males, 66 females) from July 2020 to September 2022, who had internal carotid artery stenosis of > 50% diagnosed by computed tomograph (CT) angiography or magnetic resonance (MR) angiography. Of all the patients, 151 have experienced ischemic stroke before they were diagnosed of carotid stenosis, the remaining 131 were asymptomatic with no neurological abnormalities. The common clinical symptoms of ischemic stroke include weakness or numbness of the face, arm or leg, trouble speaking and understanding, and monocular blindness. The study was approved by the ethics committee of our institution and informed consent was obtained from all patients.

The exclusion criteria were as follows:

1. Non-atherosclerotic carotid artery stenosis,
2. Carotid artery occlusion,
3. Prior carotid artery procedures,
4. Cardiogenic stroke,
5. Hemorrhagic stroke,
6. Primary intracranial diseases.

All the patients underwent brain MR examination using a 1.5 or 3.0 Tesla (T) MR scanner within 1 week of their carotid artery examination. The MRI protocol included T1WI, T2WI, FLAIR, and diffusion weighted imaging (DWI)/ADC. The imaging parameters were as follows: T1WI: repetition time (TR)/echo time (TE) = 2,000~2,400/7.6~18.0 ms; T2WI: TR/TE = 5,000~6,000/100~136 ms; FLAIR: TR/TE = 8,400~9,000/87.0~97.0 ms; and DWI/ADC: TR/TE = 4,000~5,000/77.0~85.0 ms, *b* = 0, and 1,000 s/mm^2^. Here, ms represents milliseconds and mm millimeters. Slice thickness was 5 mm and slice spacing was 1.5 mm for all the sequences.

Clinical features including sex, age, and vascular risk factors (hypertension, diabetes mellitus, hyperlipidemia, and coronary heart disease) were also recorded.

### 3.2. Deep learning architecture

**Figure 1:**
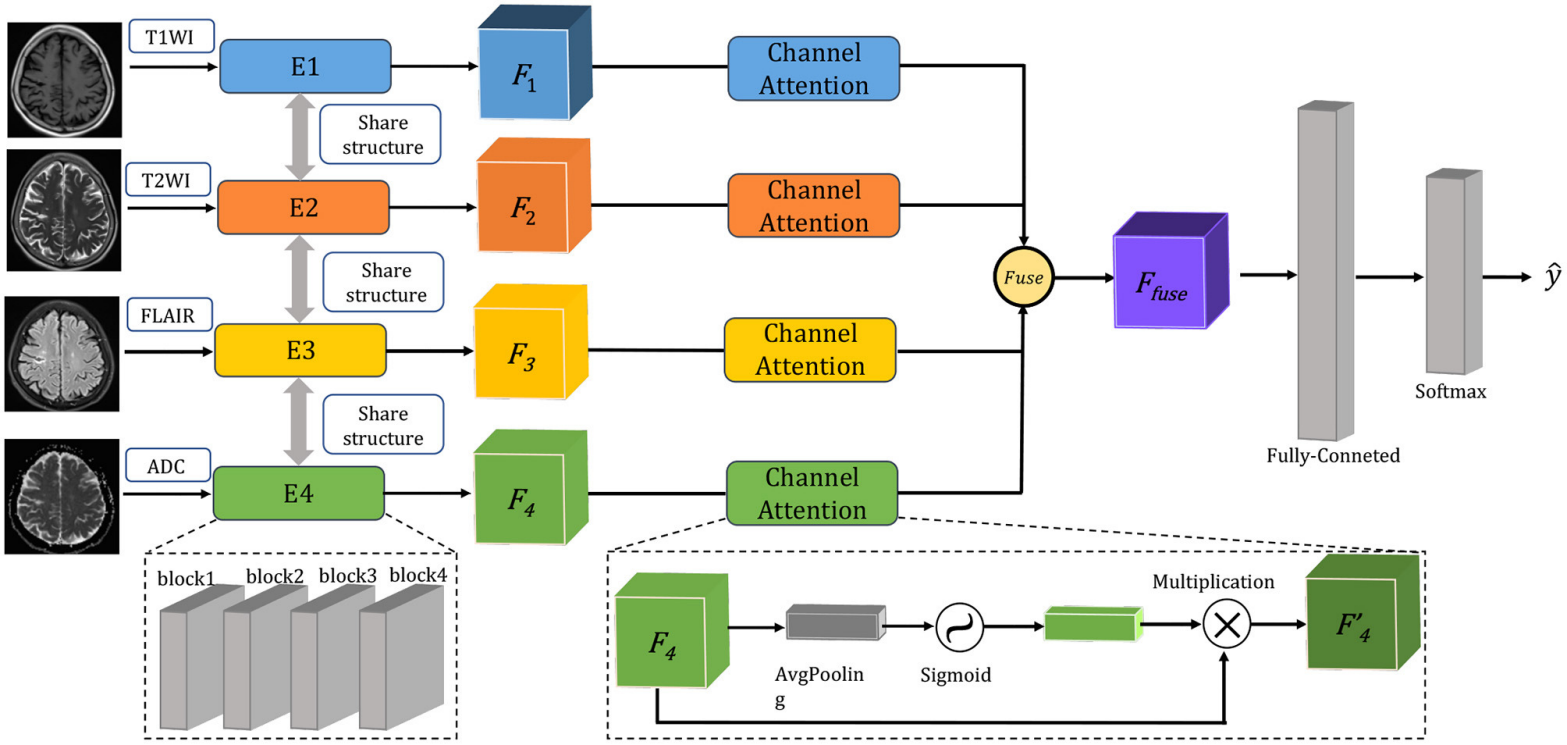
The overview of multimodal fusion containing T1WI, T2WI, FLAIR, and ADC images as input.

**Figure 2:**
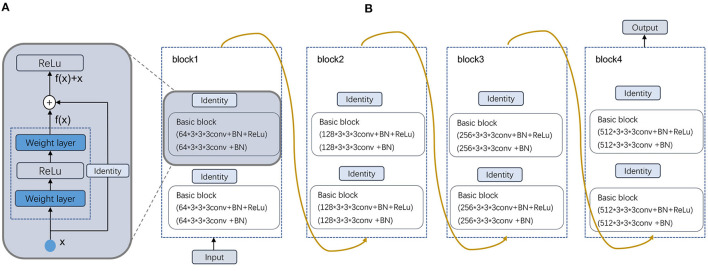
Schematic illustration of the deformed feature extraction module. **(A)** Residual block and **(B)** Four Residual layers.

In this paper, a multimodal fusion model is designed, and the overall flowchart is shown in Figure 1. Specifically, the model consists of a feature extraction encoder and a classification head for multimodal fusion, where weights are shared among the unimodal encoders and features are extracted from each unimodal input encoder, which means that all spatial locations share the same convolution kernel, which greatly reduces the number of parameter layers required for convolution. The feature extraction encoder consists of four residual blocks, and the specific flow is shown in Figure 2. The CNN model constructed in this paper mainly consists of a convolutional layer, a maximum pooling layer and a Dropout layer, and finally a fully connected layer as the output layer.

#### 3.2.1. Feature extraction module

In our dataset, the image format is digital imaging and communications in medicine (DICOM). After preprocessing, the image output size is 256 × 256 × 24. Then, after a 7 × 7 × 7 convolutional layer with 64 channels and two steps, a 3 × 3 × 3 maximum pooling layer with two steps is connected to the output. Next, four modules consisting of residual blocks are used, each using several residual blocks with the same number of output channels. The first module has the same number of channels as the number of input channels. Each subsequent module doubles the number of channels of the previous module in the first residual block and halves the height and width. For each module, two residual blocks are included, and the residual block structure is shown in Figure 2A. The residual block is to fuse the features of the (*i*)^*th*^ layer with those of the (*i*+2)^*th*^ layer, which can maximize the retention of shallow features while the network level is deepening to avoid overfitting. Four residual blocks are structured as shown in Figure 2B, and the main parameters are as follows.

For each after two convolutions of 64 convolution kernels of 3 × 3 × 3, a maximum pooling is used. Then, after two convolutions of 128 convolution kernels, a maximum pooling is used. Again, after three convolutions of 256 convolution kernels, a maximum pooling is used. This continues to repeat twice with three convolutions of 512 convolution kernels, followed by a maximum pooling; and finally after fully connected layers, the output. As the residual module deepens, the number of convolutional kernels increases, so that more channels can be obtained and different features can be extracted at a deeper level.

#### 3.2.2. Feature fusion module

To better fuse multimodal features, the feature extraction module express different modal data as low-dimensional semantic vectors and finally train a semantic similarity model, at which point the different modalities can be constrained to a unified representation space and multimodal fusion representation. Here we designed a channel attention for multimodal feature fusion. Specifically, for the image of the *m*^*th*^ modality, where *m*∈[1, 2, 3, 4]. The output features *F*_*m*_ of the feature extraction module are pooled globally in one spatial dimension to obtain a channel description of *C*×1 × 1 × 1, where *C* is the number of channels of a single modal feature. A sigmoid activation function is then used to obtain the weighting coefficients. Finally, the weight coefficients are multiplied with the corresponding input features *F*_*m*_ to obtain the new weighted features. The calculation of the weighted features is shown in the following equation:


(1) $$\begin{array}{l} {\text{F}}_{m}^{{}^{\prime }}=\left[\sigma \left(w_{m}\cdot {\text{F}}_{m}\right)\right]⊗{\text{F}}_{m}, \end{array}$$

where σ represents the sigmoid function, and *w*_*m*_ represents the parameter matrix at training time. The features of different modalities are stitched together after the maximum pooling layer. Finally, a Fully Connected (FC) layer is created in the corresponding dimension of the channel and output to the classifier to obtain the classification result.

#### 3.2.3. Loss function

In statistics, loss functions allow an evaluation of the difference values between the true value of the degree and the predicted value. The appropriate loss function usually increases the complexity of the model. There are a number of loss functions including Mean-Absolute loss function category Error (MAE), categorical cross-entropy, Mean Squared Error (MSE) and binary cross-entropy. This last lost function is used for our study, whose formula is defined as:


(2) $$Loss=\sum_{j=1}^{T}{\hat{y}}_{j}\cdot log(y_{j})+(1-{\hat{y}}_{j})\cdot log(1-{\hat{y}}_{j}),$$

where *T* denotes the number of samples. *y*_*j*_ is the true label value, and ŷ_*j*_ is the predicted probability value.

### 3.3. Machine learning architecture

On the one hand, clinical data is indispensable for physicians' diagnosis. On the other hand, for the collected clinical features, we used a ML analysis method. The data were divided in exactly the same way as the deep learning approach. We measured four common ML methods to predict stroke recurrence. These methods are as follows: random forest (Breiman, 2001), logistic regression and extreme gradient boosting (XGBoost):

1. Random forest is composed of many independent stand-alone decision trees that are individually trained on a random sample of data. The flexibility of random forest is one of its most attractive features. It can be used for recurrence detection and grouping tasks, and the overall weight of the overall weighted features on the information is obvious.
2. Logistic regression uses a collection of independent factors to predict a categorical dependent variable. Using logistic regression, the output of the categorical dependent variable is predicted. Therefore, the output must be discrete or categorical (it can be yes or no, 0 or 1, true or false, and so on).
3. The basis of XGBoost idea is to keep generating new trees. Each tree is learned based on the difference between the previous tree and the target value, thus reducing the bias of the model. Therefore, it is using multiple base learners, each of which is relatively simple to avoid overfitting. The next learner is the result of learning the previous base learner, and the difference between the model value and the actual value is continuously reduced through the learning of multiple learners.

The comparison experimental results of different classifiers are shown in Table 3.

## 4. Experiments

### 4.1. Evaluation metrics

For the analysis of the results, we used accuracy, recall, and AUC scores for evaluation. In that respect, accuracy is used to determine the performance of a classification model. It determines the ratio of the correct predictions to the total number of predictions. Recall is the ratio of true positives to the sum of true positive and false negative predictions. The area under the Receiver Operating Characteristic (ROC) is denoted as the AUC. The closer the ROC curve is to the upper left corner of the ROC curve, the more accurate the model is and the more desirable the model is. The AUC score is what determines the ability of the model to distinguish between different categories, and higher values indicate better model performance. It determines the ratio of the correct prediction to the total number of predictions.

### 4.2. Implementation details

**Figure 3:**
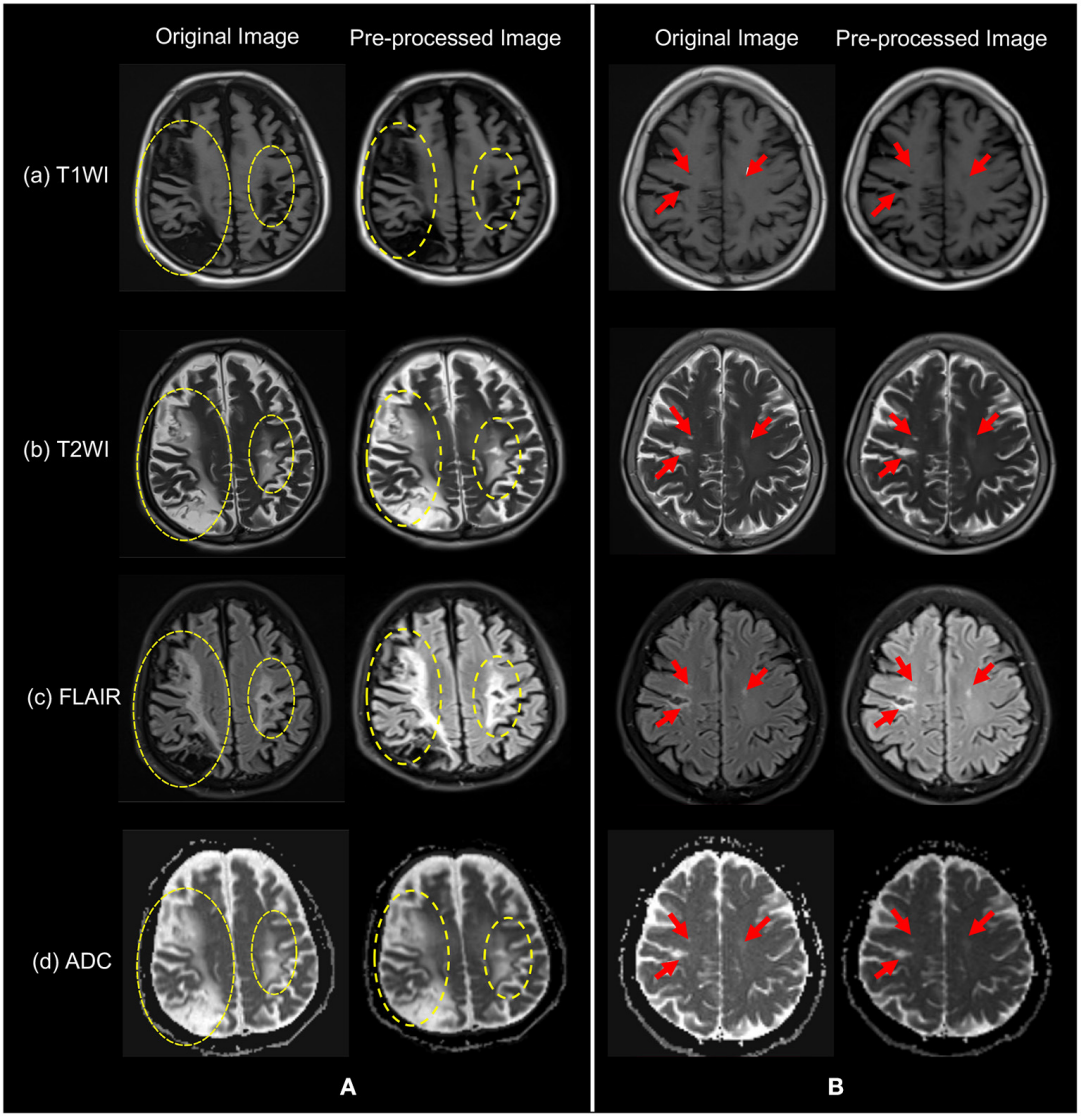
The original images of the four modalities in (a) T1WI, (b) T2WI, (c) FLAIR, and (d) ADC, and their pre-processed images. Patients in group **(A)** have yellow dashed circles indicating symptomatic areas and patients in group **(B)** have red arrows indicating symptomatic areas. Note that visually, symptoms in **(A)** are more obvious.

As our data were collected from different hospitals and different machines, the voxels of the data were not the same. In order to reduce the influence of unrelated other organ regions on feature extraction in the lesion region, thus achieving the goal of weakening data noise and improving model stability, we resampled the original unique hedge and normalized the different sized voxels in the medical images to the same size. In addition, all data were normalized to adjust the feature values of different dimensions to a similar range, and then a uniform learning rate could be used to accelerate the model training. The two sets of sample images shown in Figure 3 were original image of (a) T1WI, (b) T2WI, (c) FLAIR, and (d) ADC, and their images after pre-processing. Patients in group (Figure 3A) have a larger symptomatic area, indicated by the yellow dotted circle. Patients in group (Figure 3B) have a smaller symptomatic area, indicated by the red arrow.

For data set partitioning, in this paper, the provided training data set (four-modality MRI images) was randomly divided into training set, validation set, and test set according to the principle of positive and negative sample balance, according to whether there are bleeding symptoms for the dichotomous classification task. The main aim was to ensure that the validation samples did not overlap with the training samples, leading to the random division of the training set (*n* = 197, 70%), validation set (*n* = 42, 15%), and test set (*n* = 43, 15%).

Due to the small amount of data obtained from the hospital, less coverage of imaging features may lead to over-fitting problems. The model may not apply to other untrained data. Therefore, data expansion methods including zooming in, zooming out and moving this study compensated for the lack of data. The final softmax classifier of the convolutional neural network had final nodes that predicted the probability of each class based on the features extracted from the network. The models were implemented using PyTorch and trained by the Adam (Kingma and Ba, 2014) optimization algorithm. The models were trained for 300 epochs. All models were trained from scratch using an initial learning rate of 1 × 10^−4^. The final loss function was computed for each task for all samples in the batch with known base fact labels and averaged to a global loss, which was then back-propagated through the models to a predictive loss for the final labels. These parameters were well-verified in the industry empirically.

### 4.3. Analysis on single modality

**Figure 4:**
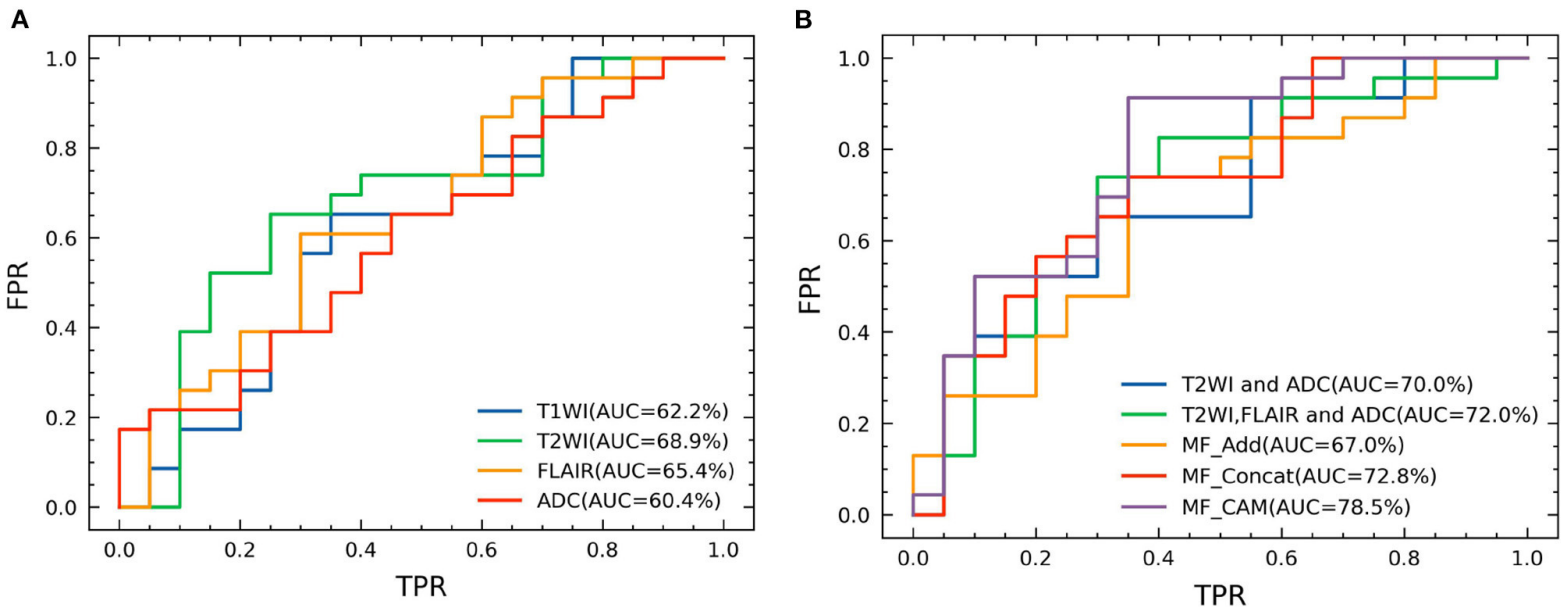
ROC curves of our network and compared methods. **(A)** Single modality and **(B)** Multi modality fusion.

Based on the same data partitioning method and the same experimental setup as above, we first performed feature extraction and classification of the unimodal MRI images. The experimental results are reported in Table 1. In the single-modality experiments, the AUC metrics of the four different modalities reached 62.2, 68.9, 65.4, and 60.4%, respectively, with the T2WI modality being 8.5% higher than the ADC modality AUC metric. Comparing the accuracy classification performance of the four different modalities longitudinally, the ADC modality was relatively poor, 11.6% lower than the better-performing FLAIR. The ROC curves based on four single modality image classifications are shown in Figure 4A.

**Table 1:** Comparison of classification performance based on different modalities.

**Modality**	**AUC (%)**	**Accuracy (%)**	**Recall (%)**
T1WI	62.2	60.5	78.3
T2WI	**68.9**	60.5	**100**
FLAIR	65.4	**65.1**	87.0
ADC	60.4	53.5	69.7

The bold values represent the best values.

### 4.4. Analysis on multi-modal fusion

In order to verify the effectiveness of data fusion of different modes, we designed a fusion experiment of different modes, and the experimental results are shown in Table 2. Compared with the experimental results in Table 1, the classification effect of multimodal fusion is improved, which verifies the theory that different modalities can provide richer information. In addition, for AUC measurement, the fusion result of T2WI and ADC mode is lower than the fusion classification result of T2WI, FLAIR and ADC, which indicates that FLAIR mode provides richer features.

**Table 2:** Comparison of classification performance based on different modalities.

**Modality**	**AUC (%)**	**Accuracy (%)**	**Recall (%)**
T2WI and ADC	70.0	65.1	82.6
T2WI and FLAIR and ADC	72.0	67.4	60.9
MF_CAM (ours)	**78.5**	**76.7**	**87.0**

The bold values represent the best values.

In addition, to verify the effectiveness of different fusion methods, we designed three groups of different fusion experiments, all of which fused the features of four modes. Table 3 shows the results of ablation research. Specifically, MF_Add directly adds the features of different modes, and MF_Concat splices the features of different modes through channels. In this paper, MF_CAM is used. Before feature fusion, the attention mechanism of single-mode images is increased, and then the features of key areas of single mode are enhanced and then spliced by channels. From the experimental results, the performance of MF_Add is the worst, which shows that the feature addition fusion method is not a desirable choice. In addition, MF_Concat does not fully consider the importance of different modal features, and direct splicing may have feature redundancy. In contrast, the model MF_CAM with attention mechanism has achieved better fusion classification results. It is verified that the attention mechanism designed in this paper can help the model to focus on the same symptomatic area after fusing the features of different modalities, so feature fusion can obtain richer and more comprehensive tumor features of different modalities, which greatly improves the classification results. ROC curves based on four kinds of multimodal image classification are shown in Figure 4B.

**Table 3:** Comparison of classification performance based on different modalities.

**Modality**	**AUC (%)**	**Accuracy (%)**	**Recall (%)**
MF_Add	67.0	60.5	56.5
MF_Concat	72.8	67.4	60.9
MF_CAM (ours)	**78.5**	**76.7**	**87.0**

The bold values represent the best values.

### 4.5. Effectiveness of machine learning

The results of classification based on clinical features using three different machine learning algorithms (RF, logistic regression, and XGBoost) were shown in Table 4. The AUCs of the three algorithms were 50.6, 64.8, and 66.8%, respectively. Among them, the value of AUC of XGBoost classification algorithm reached 66.8%, which was the highest among the three classification algorithms, and the classification effect was average compared with the deep learning methods based on image data in Table 1. Therefore, compared with clinical features, image data can provide more abundant information, which is indispensable for doctors' diagnosis.

**Table 4:** A quantitative comparison of the effectiveness of different machine learning algorithms based on clinical data.

**Methods**	**AUC (%)**	**Accuracy (%)**	**Recall (%)**
Random forest	50.6	54.8	63.6
Logistic regression	64.8	**64.3**	63.6
XGBoost	**66.8**	59.5	**72.7**

The bold values represent the best values.

## 5. Discussion

Carotid atherosclerotic stenosis-related cerebral ischemic injury, especially infarctions and white matter hyperintensities, can be easily assessed with routine brain MRI. The signal abnormalities of cerebral ischemic injury on MRI is far more common than clinical cardiovascular risk factors. These imaging findings are strong, independent risk factors for future symptomatic stroke. Meta-analysis of a large amount of studies showed that presence of bran infarctions and extensive white matter hyperintensities burden were associated with higher risk of ischemic stroke (hazard ratio, 2.18 and 2.39, respectively) (Debette et al., 2019). In this paper, the AUC value of the classification experiment based on single mode can reach 68.9% (T2WI), and the AUC value of the experiment based on the fusion of T1WI, T2WI, FLAIR, and ADC can reach 72.8%. To the best of our knowledge, this is the first study that used brain MRI data to build a prediction model using deep learning algorithms. The performance of the model is better than the Meta-analysis also used brain MRI data and the revised FSRS which is widely used in clinic (Flueckiger et al., 2018; Debette et al., 2019). The multiple resources of MRI data partly ensured the reliability of the model in real-world clinical situations.

## 6. Conclusion

In this paper, we first collected 282 cases of carotid artery stenosis, each of which contained four different modes of brain MRI images, namely: T1WI, T2WI, FLAIR, and ADC. In this way, a multi-modal data set is constituted for judging whether patients had symptoms or not. In addition, we propose a multi-modal fusion learning classification network. Our method can fuse multi-modal image features, effectively enhance the model representation ability, and effectively improve the classification performance compared with single-modality MRI images. As they are widely used in clinical practice, our current study included only routine brain MRI sequences. In the future, the data of perfusion weighted imaging, also susceptibility weighted imaging and functional MRI could be added to a comprehensive model to provide more imaging information.

## Data availability statement

The raw data supporting the conclusions of this article will be made available by the authors, without undue reservation.

## Author contributions

PL: study concept and design, analysis and interpretation of data, drafting/revising the manuscript for content, and statistical analysis. JY and JW: design the experimental methods, investigation and the verification of experimental results, and the writing of the manuscript. YG and QT: analysis and interpretation of data. BM: investigation, verification, writing—review, and editing. JL and JZ: study concept and design and drafting/revising the manuscript for content. All authors contributed to the article and approved the submitted version.
